# A novel angiogenesis-based molecular signature related to prognosis and tumor immune interactions of pancreatic cancer

**DOI:** 10.3389/fcell.2022.1001606

**Published:** 2022-10-06

**Authors:** Weiyu Ge, Daiyuan Shentu, Yongchao Wang, Yanling Wang, Shengbai Xue, Ming Yue, Tiebo Mao, Xiaofei Zhang, Haiyan Xu, Shumin Li, Jingyu Ma, Jiayu Yao, Jiujie Cui, Liwei Wang

**Affiliations:** State Key Laboratory of Oncogenes and Related Genes, Shanghai Cancer Institute, Department of Oncology, Renji Hospital, School of Medicine, Shanghai Jiao Tong University, Shanghai, China

**Keywords:** angiogenesis, pancreatic cancer, prognosis, tumor immune microenvioronment, molecular signature

## Abstract

Angiogenesis, a hallmark of cancer, is related to prognosis, tumor progression, and treatment response. Nevertheless, the correlation of angiogenesis-based molecular signature with clinical outcome and immune cell infiltration has not been thoroughly studied in pancreatic cancer. In this study, multiple bioinformatics methods were combined to evaluate prognosis, immune cell infiltration, and the alterations of angiogenesis-related genes (ARGs) in PC samples, and further establish a novel angiogenesis-related gene signature. Moreover, the protein and mRNA expression levels of four angiogenesis risk genes were determined by Human Protein Atlas (HPA) database and qPCR analysis, respectively. Here, we recognized two distinct angiogenesis subtypes and two gene subtypes, and revealed the critical roles of ARGs in the tumor immune microenvironment (TIME), clinical features, and prognosis. Consequently, we established an ARGs score to predict prognosis and therapeutic response of PC patients, and validated its robust predictive ability. Additionally, the ARGs score was markedly associated with clinical outcomes, tumor mutation burden (TMB), and chemotherapeutic drug sensitivity. In brief, our findings imply that the ARGs score is a robust prognostic indicator and may contribute to the development of effective individualized therapies for PC.

## 1 Introduction

Pancreatic cancer (PC) is among the most lethal malignancies worldwide (Siegel et al., 2021), and is characterized by high invasiveness, recurrence, and therapy resistance (Kamisawa et al., 2016). For the majority of patients with advanced PC, treatment options are limited. In recent years, despite varied targeted drugs and chemotherapeutic regimens having been developed, therapy effects remain unsatisfactory, with a 5-year overall survival rate slightly more than 10% (Mizrahi et al., 2020) and a median survival time of 7 months (Luo et al., 2020; Stornello et al., 2020; Khalaf et al., 2021). In previous studies, the traditional histological classification of PC was proved to be limited due to its highly heterogeneous and complex characteristics (Whatcott et al., 2015). As a result, study of molecular subtypes has become increasingly important for guiding therapy. In addition, there is now evidence that immunotherapy combination with chemotherapy, such as PD-1 checkpoint blockade combination with gemcitabine, provides additional treatment opportunities for PC patients, and that certain types of patients may benefit from them (Wainberg et al., 2019; Schizas et al., 2020). Thus, valuable biomarkers are essential for classifying patients with distinct features into different groups and predicting the impact of immunotherapy.

Angiogenesis, namely, the formation of newly formed blood vessels from pre-existing ones, is one of the critical factors supporting tumorigenesis and metastasis of multiple cancers, including PC(Baeriswyl and Christofori, 2009; Zhang et al., 2018). PC presents abundant deposition of fibrotic stroma and easily invades lymphovascular system (Whatcott et al., 2015; Kamisawa et al., 2016). There is also evidence that tumor immune microenvironment (TIME) and angiogenesis appear to interact cross-talk, for example, angiogenic cytokines mediate immunosuppression by activating suppressing immune cells (such as Tregs and tumor-associated macrophages) (Griesmann et al., 2017; Ren et al., 2018; Saito et al., 2018; Rahma and Hodi, 2019). There are several proven therapeutic strategies for inhibiting angiogenesis in solid tumors. Nevertheless, PC patients who receive anti-angiogenic therapies do not experience satisfactory outcomes (El-Kenawi and El-Remessy, 2013; Ramjiawan et al., 2017; Viallard and Larrivée, 2017; Yu et al., 2019).

Therefore, a thorough understanding of properties of ARGs mediated immune cell infiltration in PC tumor microenvironment (TME) may shed light on guiding targeted therapy and immunotherapy. This study comprehensively estimated the expression profiles of ARGs and their influence on clinicopathological features, prognosis, TIME, and therapeutic response of PC patients in two independent cohorts. Firstly, we identified two distinct angiogenesis subtypes in PC. Subsequently, we evaluated the molecular features, prognosis, and immune cell infiltration of the identified angiogenesis subtypes and two gene subtypes. Furthermore, we established an ARGs score to predict clinical outcome of PC patients and chemotherapeutic effects. Finally, we validated the protein and mRNA expression level of four angiogenesis risk genes. These findings indicate that ARGs score is a robust prognostic indicator, and we hope our study will contribute to exploring more effective therapeutic strategies for PC.

## 2 Materials and methods

### 2.1 PC data sets and preprocessing

The normalized transcriptome data of normal pancreatic tissue in GTEx (n = 167) cohort, tumor tissue in TCGA-PAAD program (n = 160) and ICGC-PAAD repository (n = 101) and corresponding clinicopathological information of PC were downloaded from the UCSC Xena data portal (https://xenabrowser.net/datapages/), which integrates all the data from The Cancer Genome Atlas (TCGA), Genotype-Tissue Expression (GTEx) and International Cancer Genome Consortium (ICGC) in June 2021) Somatic mutation data from TCGA data portal (https://portal.gdc.cancer.gov/) sorted in the form of Mutation Annotation Format (Saito et al., 2018) were analyzed. We excluded samples from patients with a deficiency of important survival or clinicopathological information. Then, the normalized gene expression data (FPKM values) of all cohorts were performed to subsequent analysis, the R (version 3.6.3) and R Bioconductor packages were used for all data analysis.

### 2.2 Consensus clustering analysis for prognostic ARGs

Initially, the univariate Cox regression analysis was employed to assess the prognostic values of 36 ARGs obtained from the MSigDB Team (Hallmark Gene set) in PC patients, then, *p* < 0.05 was selected as a screening threshold, and 11 prognostic ARGs were screened out with univariate Cox analysis (*p* < 0.05). Based on the expression level of these ARGs, 428 PC cases from TCGA, ICGC, and GTEx cohorts were divided into two distinct subtypes by the consensus clustering analysis with “ConsensusClusterPlus R package” (Seiler et al., 2010; Sabah et al., 2021).

### 2.3 Functional and pathway enrichment analysis

The performances of cancer hallmarks in the TCGA and GTEx cohorts were evaluated using the single sample gene set enrichment analysis (ssGSEA) algorithm (R package “gsva”) based on normalized transcriptome data and hallmark gene sets of “h.all.v7.5.1. symbols.gmt” from the Molecular Signatures Database (MSigDB) (Barbie et al., 2009; Liberzon et al., 2011). The gene sets of “c2. cp.kegg.v7.5″ were also retrieved from the MSigDB database to perform gene set variation analysis (GSVA) and gene set enrichment analysis (GSEA) enrichment analysis. The R package clusterProfiler was applied for functional annotation of the ARGs. And *p* < 0.05 was supposed to be valuable in gene ontology (Yu et al., 2012).

### 2.4 Correlations of molecular patterns with the clinical properties and prognosis of PC

To assess the clinical significance of the two subtypes classified by consensus clustering, we compared the association of molecular patterns with clinical properties and prognosis. The clinical properties included age (≥65 and <65 years), gender (male and female), tumor location (head and tail/body), TNM stage (stage I–IV), KRAS mutation status (abnormal and normal), and TP53 mutation status (abnormal and normal). Furthermore, the differences in overall survival (OS) among two molecular patterns were estimated by Kaplan-Meier analysis with the “survival” and “survminer” packages (Rich et al., 2010).

### 2.5 Associations of molecular subtypes with TME in PC

The ESTIMATE algorithm (Meng et al., 2020) was applied to evaluate the immune and stromal scores of each PC patient. Moreover, the proportions of 22 immune cell subtypes of each PC sample were assessed with the CIBERSORT algorithm (Chen et al., 2018). Besides, the infiltrating scores of TME in each PC were also identified by a single-sample gene set enrichment analysis (ssGSEA) algorithm (Huang et al., 2021).

### 2.6 Identification of gene clusters and functional enrichment analysis based on two angiogenesis subtypes

The differentially expressed genes (DEGs) and functional annotation among the angiogenesis subtypes were distinguished by the empirical Bayesian approach with the “limma” R package (Ritchie et al., 2015). The DEGs were screened out with the criteria of |log2-fold change (FC)| ≥0.5 and *p*-value < 0.05 (Zhang et al., 2020). Subsequently, the GO and KEGG analysis was performed to further thoroughly investigate the potential functions of angiogenesis subtypes-related DEGs with the “clusterProfiler” package (Yu et al., 2012). Moreover, the univariate Cox regression analysis based on these DEGs was conducted to quantify the OS-related DEGs in patients with PC. Then, based on the expression of prognostic DEGs, all patients from TCGA cohort were divided into different subtype groups (gene cluster A and gene cluster B) for further clustering analysis with a consensus clustering algorithm.

### 2.7 Construction of the prognostic angiogenesis signature

Firstly, univariate Cox regression evaluated the prognostic values of 36-angiogenesis genes from the MSigDB Team in patients with PC, and *p* < 0 .05 was selected as the threshold for filtering, 11 prognostic angiogenesis-related genes (ARGs) were selected. Next, we further performed a multivariate Cox regression analysis and the penalized Cox regression model with least absolute shrinkage and selection operator (LASSO) (Gao et al., 2010) on 11 prognostic ARGs, four of which (TNFRSF21, CCND2, JAG1and SPP1) and their corresponding coefficients were identified as independent predictive factors. Finally, an angiogenesis gene signature was conducted based on these hub ARGs, defined as the ARGs score.

The ARGs score was calculated as follows:

ARGs score=∑(Coefficient×Expression)

Based on the median risk score, all PC patients were classified into low- and high-ARGs score groups.

### 2.8 Cell culture

Human PC cell lines (BXPC-3, CFPAC-1, PANC-1) and one normal pancreatic duct cell line (HPNE) were obtained from the Chinese Academy of Science (Shanghai, China) and cultured in RPMI 1640 medium and DMEM medium (HyClone) supplemented with 100 U/ml penicillin-streptomycin (Corning, NY, United States), 10% fetal bovine serum (Gibco, NY, United States) at 37°C with 5% CO_2_.

### 2.9 Protein levels of angiogenesis risk genes in the Human Protein Atlas database

The Human Protein Atlas (HPA) database (https://www.proteinatlas.org/) consists of all the human proteins in cells, normal and tumor tissues using integration of multiple omics technologies, such as immunofluorescence and immunohistochemistry (Uhlén et al., 2015). So, we use HPA online tool to analyze protein levels of specified genes in normal and tumor tissues by immunohistochemistry data.

### 2.10 Association between angiogenesis risk genes and metastasis

HCMDB (Human Cancer Metastasis Database, http://hcmdb.i-sanger.com/index) is an integrated database designed to store and analyze large scale expression data of cancer metastasis. Which is freely accessible to the research community query cross-platform transcriptome data on metastases. A total of 124 previously published transcriptome datasets were collected from GEO and TCGA. HCMDB is developed and maintained as a useful resource for building the systems-biology understanding of metastasis (Zheng et al., 2018). In the present study, the HCMDB database was used to detect the metastatic potential of specific gene. Here, we explored the significance of angiogenesis risk genes in PC metastasis by HCMDB.

### 2.11 Quantitative real-time PCR reaction (qRT-PCR)

Cellular total RNA was extracted with an E.Z.N.A total RNA Kit I (Omega Bio-Tek, Inc., Norcross, GA, United States). The RNA purity was detected using NanoDrop 2000 spectrometer (Thermo Fisher Scientific, Waltham, MA). The cDNA was synthesized with a PrimerScript RT reagent Kit (Takara, Ostu, Shiga, Japan) for reverse transcription reactions. qRT-PCR was performed to detect the expression levels of the four genes using the SYBR Premix Ex Taq kit (Takara) with Roche LightCycler480 PCR instrument according to the manufacturer’s protocol. The primers used in our study were listed in Supplementary Table S1. β-ACTIN was used as an internal control. The relative mRNA levels were calculated based on 2−ΔΔCt method.

### 2.12 Clinical association and classification analysis of the prognostic ARGs score

Chi-square tests were applied to search for the associations between the ARGs score and the clinical properties. In addition, we performed a classification analysis to explore whether the ARGs score remains predictive and reliable in distinct subtypes based on several clinical variables.

### 2.13 Evaluation of immune status and tumor mutation burden (TMB) between the high-and low-ARGs score groups

To assess the fractions of tumor-infiltrating immune cells (TIICs) and the expression level of immune checkpoint (ICP) among the different ARGs score subtypes. We explored the associations between the proportions of 21 TIICs and four hub ARGs in the different ARGs score groups. Furthermore, based on the median value, all PC patients were classified into high and low tumor mutation burden (TMB) groups, we further identified the associations of ARGs score with TMB and survival. Next, to compare the somatic mutations of PC patients between high- and low-ARGs score groups, the mutation annotation format (Saito et al., 2018) from the TCGA database was analyzed with the “maftools” R package (Mayakonda et al., 2018).

### 2.14 Drug sensitivity analysis

To investigate differences in the therapeutic impacts of chemotherapeutic drugs for PC patients of the two groups, we used the “pRRophetic” package to estimate the half-maximal inhibitory concentration (IC50) of chemotherapeutic drugs commonly used in the treatment of tumors, which is based on drug-sensitive data from Genomics of Drug Sensitivity in Cancer dataset (GDSC, https://www.cancerrxgene.org/) (Geeleher et al., 2014).

### 2.15 Statistical analyses

The Wilcoxon test was applied to analyze the difference between the groups. The correlation tests were conducted by Spearman analyses. Kaplan-Meier survival analysis were performed to draw survival curves by the log-rank tests. And the “ggplot2” R software package is used for principal component analysis (PCA). The Time-dependent receiver operating characteristic (ROC) curves for 1-, 2-, and 3-year survivals were performed to validate predictive capability of ARGs score and other clinical variables with the R package ‘survivalROC’. The R 3.6.3 software and its corresponding packages are applied to process, analyze, and present the data. While comparing between groups, *p* < 0.05 was deemed to be statistically significant.

## 3 Results

### 3.1 Angiogenesis as a cancer hallmark is hyperactivated in PC

**FIGURE 1:**
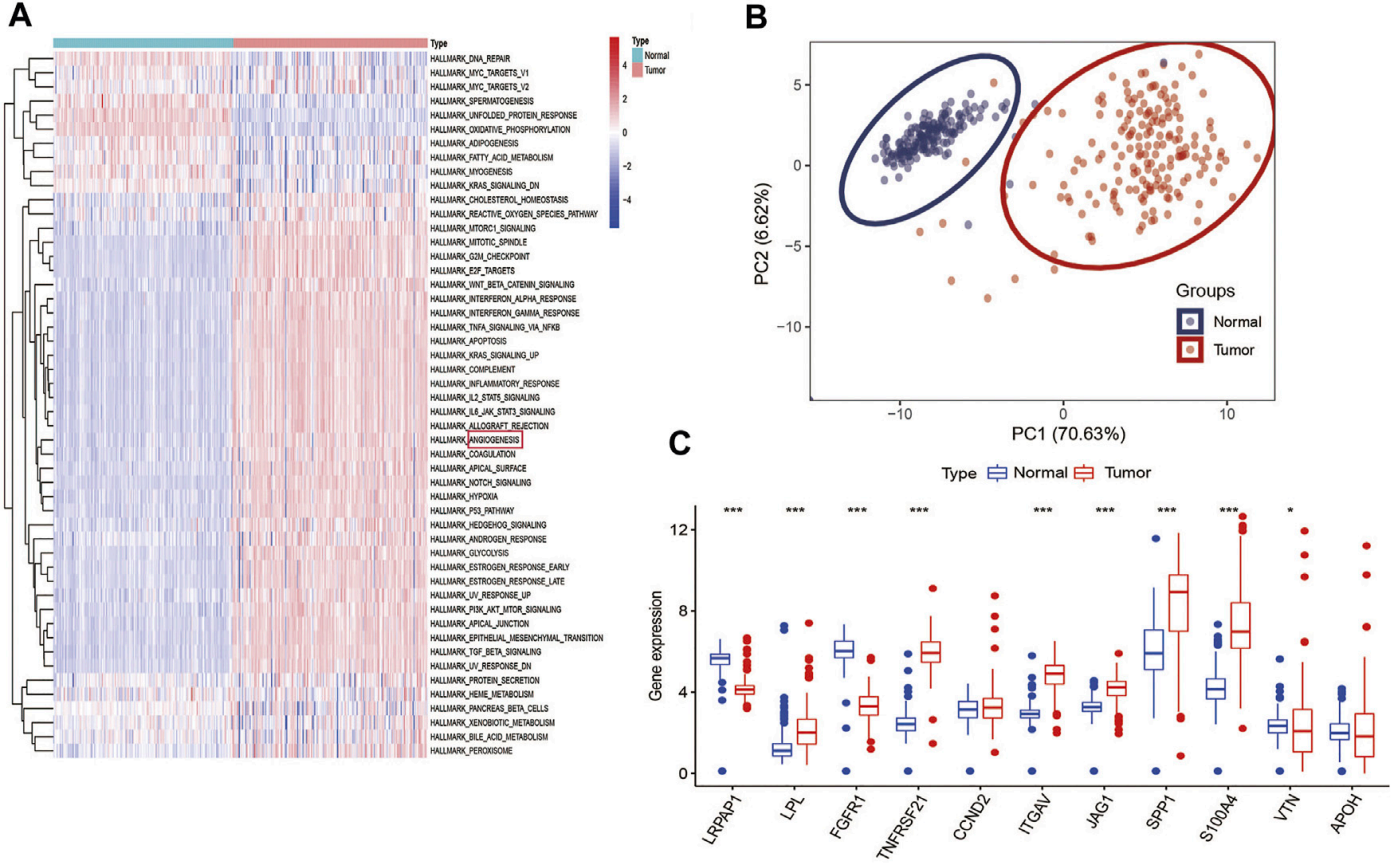
Angiogenesis as a cancer hallmark is hyperactivated in PC: **(A)**, GSVA analysis demonstrated that angiogenesis is hyperactivated in PC among various hallmarks of cancer; **(B)** PCA analysis demonstrated that PC and normal pancreas samples were evidently separated as two distinct groups with the 11 prognostic ARGs expression matrix; **(C)** Expression distributions of 11 ARGs between normal and PC tissues. ***p < 0.001, **p < 0.01, *p < 0.05 and not significant (*p* > 0.05) by repeated measures with the Wilcoxon test.

Among 50 cancer hallmarks gene sets, GSVA analysis indicated that compared with normal specimens (GTEx), 34 cancer hallmarks are aberrantly hyperactivated in tumor PC specimens from TCGA cohort, such as angiogenesis, hypoxia, epithelial-mesenchymal transition (EMT) and apoptosis (Figure 1A). Supplementary Table S2 presented detailed information on the 160 PC patients from TCGA cohort. To explore the classification of angiogenesis in tumor and normal samples, the 11 prognostic ARGs (*LRPAP1, LPL, FGFR1, TNFRSF21, CCND2, ITGAV, JAG1, SPP1,* S100A4, *VTN*, and *APOH*) were analyzed using consensus clustering analysis, and the PCA analysis confirmed the excellent intergroup distribution (Figure 1B). The correlation coefficients of the 11 prognostic ARGs are provided in Supplementary Table S3. Next, we observed that the expression of almost ARGs are significantly different between tumor samples and normal samples, some ARGs (*LPL, TNFRSF21, ITGAV, JAG1, SPP1,* and S100A4) were up-regulated in the tumor samples (Figure 1C), however, *LRPAP1* and *FGFR1* were down-regulated in the tumor samples, indicating the potential role of these ARGs in PC tumor development.

### 3.2 Identification of angiogenesis subtypes in PC

**FIGURE 2:**
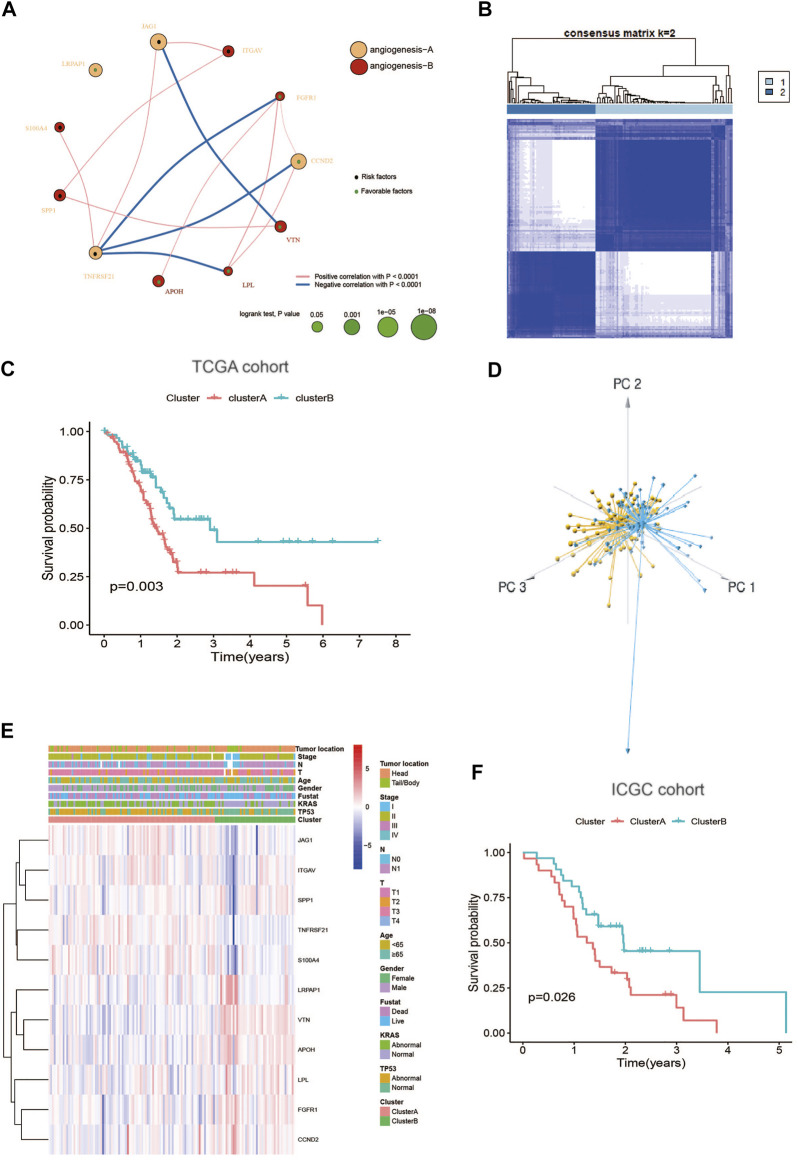
Identification of angiogenesis subtypes in PC: **(A)** A network of Interactions among ARGs in PC, the size of the circle represents the impact of each angiogenesis gene on the prognosis, the *p* value calculated by log-rank test. Green dots in the circle represent protective factors and black dots represent risk factors. Links between genes represent their interactions, blue lines represent positive correlations, red lines represent negative correlations, and the thickness of the lines represents the strength of the correlation between them; **(B)** Consensus matrix heatmap defining two clusters (k = 2) and their correlation area; **(C)** Kaplan–Meier plot of OS by angiogenesis clusters for PC patients in the TCGA cohort (*p* = 0.003, Log-rank test); **(D)** PCA analysis showing two distinct subtypes of 11 angiogenesis genes in TCGA cohort; **(E)** Heatmap plot of 11 angiogenesis genes expression with clinical characteristics in PC from TCGA cohort. **(F)** Kaplan–Meier plot of OS by angiogenesis clusters for patients in the ICGC cohort.

The detailed flowchart of this work is shown in Supplementary Figure S1. To comprehensively understand the expression profile of ARGs in PC, the systematic genetic pattern of ARGs interactions, regulator associations, and their clinical significance in PC patients were displayed in an angiogenesis network (Figure 2A and Supplementary Table S4). Moreover, the consensus clustering analysis and PCA display that k = 2 appeared to be an optimal choice for dividing the entire cohort into angiogenesis cluster A (n = 97) and B (n = 63) (Figures 2B,D; Supplementary Figure S2). And patients in angiogenesis cluster B show a better OS than angiogenesis cluster A (log-rank test, *p* = 0.003; Figure 2C). Additionally, angiogenesis cluster A is correlated with “higher mortality”, “higher KRAS mutations " and " higher TP53 mutations” compared with angiogenesis cluster B (Figure 2E). Similarly, the ICGC cohort was applied to verify the reliability of the clustering, and two different subtypes were clearly identified again (Supplementary Figure S3). And diverse differences in survival were also shown in the two clusters (Figure 2F), further confirming the repeatability of the clustering.

### 3.3 Characteristics of tumor immune interactions and biological function in the angiogenesis subtypes

**FIGURE 3:**
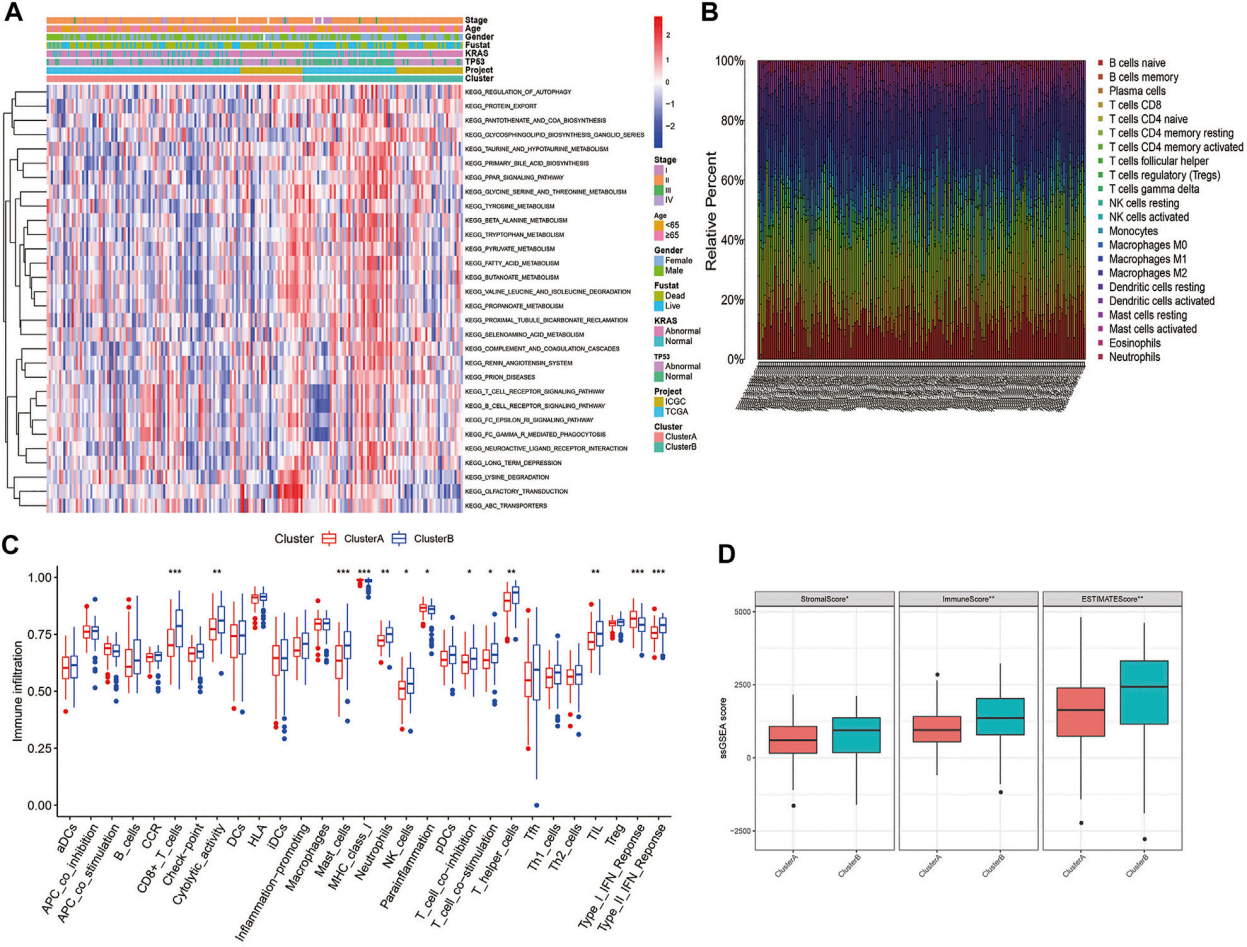
Correlations of TIME and two PC subtypes: **(A)** GSVA analyzed the biological pathways of two angiogenesis subtypes in all samples from TCGA and ICGC cohorts; **(B)** The relative percentage of 22 subpopulations of immune cells in PC; **(C)** 29 TIME cells infiltration abundance of two angiogenesis subtypes; **(D)** Correlations between the two angiogenesis clusters and TME score. ***p < 0.001, **p < 0.01, *p < 0.05 and not significant (p > 0.05) by repeated measures with the Wilcoxon test.

GSVA enrichment analysis displays that angiogenesis cluster B was significantly enriched in immune-activated pathways (T and B cell receptor signaling pathway) (Figure 3A; Supplementary Table S5). To further investigate the differences in the infiltrating immune cells among the two subtypes, the enrichment score of the 29 immune cell subsets and the relative percentage of the 22 infiltrating immune cell subsets of the two clusters in each PC patient were assessed using ssGSEA and CIBERSORT analysis. As shown in Figures 3B,C, the angiogenesis cluster B was significantly associated with tumor immune activation due to more immune cell infiltrations such as CD8+T cells, cytolytic activity, mast cells, MHC class I, neutrophils, NK cells, T cell co-inhibition, T cell co-stimulation, T helper cells, TIL, Th2 and type II IFN response, as well as with higher TME scores (Figure 3D). In contrast, para-inflammation and type I IFN response showed higher infiltration in angiogenesis cluster A. Our findings suggest that the immune cell activation is a feature of the angiogenesis cluster B.

### 3.4 Identification of angiogenesis gene clusters based on DEGs

To thoroughly investigate the potential biological function of each angiogenesis subtype, we obtained 234 angiogenesis clusters-associated DEGs with the “limma” package and conducted functional enrichment analysis. To thoroughly investigate specific adjustment mechanisms of each angiogenesis subtype, we obtained 332 angiogenesis clusters-associated DEGs (Supplementary Table S6) with the “limma” package and conducted functional enrichment analysis. Then, a consensus clustering method was utilized to separate patients into two distinct gene clusters (gene cluster A and B) with different prognosis and clinical features on the basis of DEG (Song et al., 2021; Zhang et al., 2021; Qing et al., 2022; Zhang et al., 2022). Consistent with the expected results of the angiogenesis subtypes, we observed significant differences in ARGs expression levels (Supplementary Figure S4).

### 3.5 Establishment and validation of the prognostic ARGs score

The ARGs score was constructed based on four key ARGs (*TNFRSF21, CCND2, JAG1,* and *SPP1*), and the correlations between the ARGs score and the expression of these biomarkers are provided in Supplementary Table S7. Among these biomarkers, *TNFRSF21, JAG*, and *SPP1* are biomarkers with high ARGs score coefficient, while *CCND2* served as a protective biomarker in this model.

Eventually, the ARGs score was accessed as described:

$$\begin{array}{ll} ARGs\;score= & \left(1.98\times expression\;of\;TNFRSF21\right)+\left(-0.59\times expression\;of\;CCND2\right)\\ & +\left(1.69\times expression\;of\;JAG1\right)+\left(0.55\times expression\;of\;SPP1\right) \end{array}$$

**FIGURE 4:**
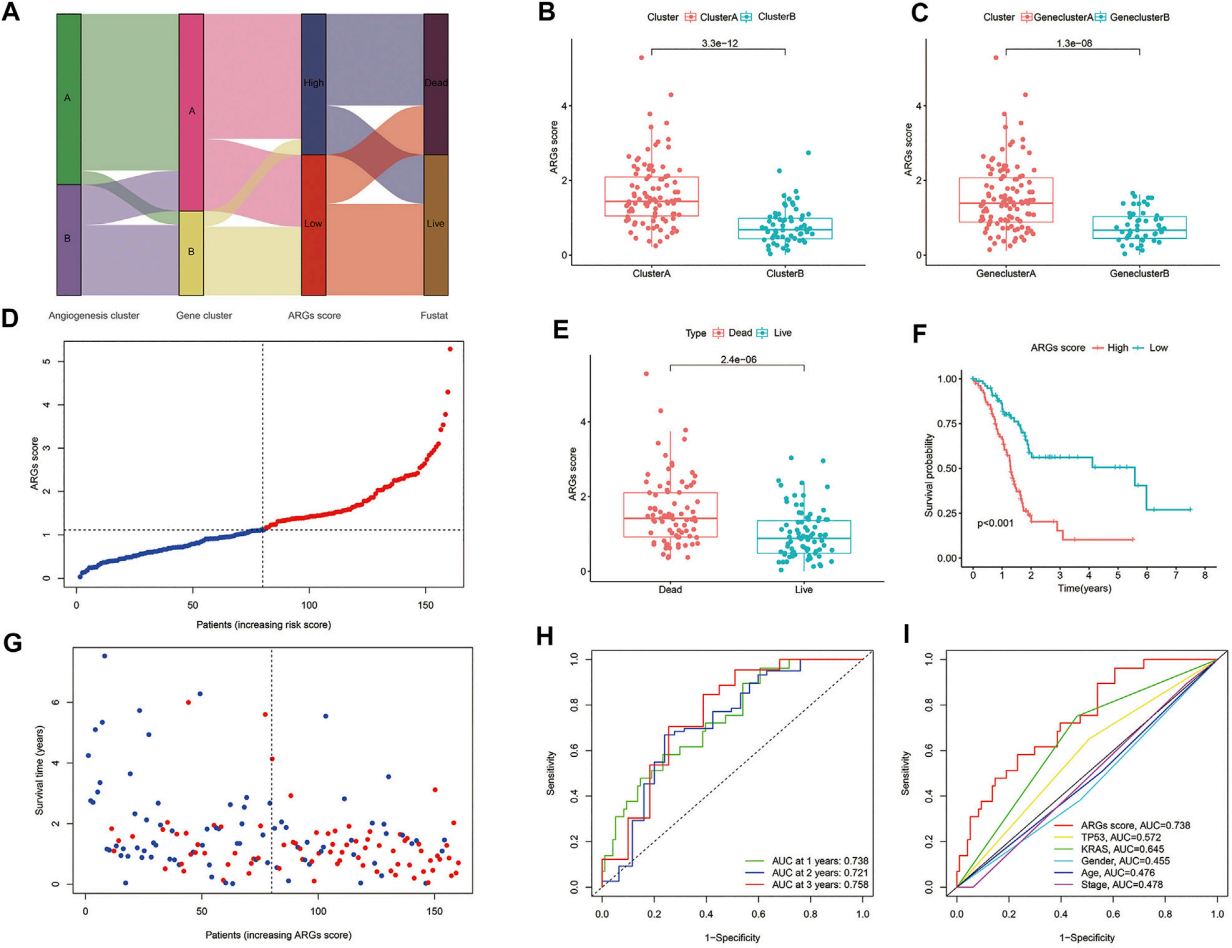
Construction of angiogenesis signature: **(A)** Sankey diagram of subtype distributions in groups with different ARGs scores and survival outcomes; **(B)** Differences in ARGs score between gene subtypes; **(C)** Differences in ARGs score between angiogenesis clusters; **(D,G)** Ranked dot and scatter plots showing the ARGs score distribution and patient survival status; **(E)** ARGs score was significantly elevated in dead patients; **(F)** Kaplan-Meier curves were used to analyze the survival of patients with high- and low- ARGs score in PC patients; **(H)** ROC curves were used to predict the sensitivity and specificity of 1-, 2- and 3-year survival according to the ARGs score; **(I)** tROC curves of the nomograms compared for 1-, 2- and 3-year OS in PC, respectively.

Figure 4A displays the distribution of patients in the two angiogenesis clusters, two gene clusters, and two ARGs score groups. In addition, we observed a significant difference in ARGs score between the two angiogenesis subtypes and also between the two gene subtypes (Figures 4B,C). More importantly, compared to angiogenesis cluster B and gene cluster B, angiogenesis cluster A and gene cluster A had a significantly higher ARGs score, implying that a low ARGs score may be closely linked to immune cell activation. The risk distribution of ARGs score revealed that with the increase of ARGs score, survival time decreased and mortality rose (Figure 4D). And a heatmap of four hub ARGs was presented in Supplementary Figure S5. Figure 4E reveals that ARGs scores were apparently elevated in dead patients compared with living patients during follow-up. Based on the survival analysis mentioned above, we found that higher ARGs scores were associated with worse survival (Figures 4F,G). The AUCs of 1-, 2-, and 3-year OS were 0.732, 0.721, and 0.758, respectively (Figure 4H). Moreover, we assessed the AUC values of these clinical variables (age, gender, TNM stage, KRAS mutation status, and TP53 mutation status) and ARGs score for predicting OS at 1-, 2-, and 3-year. The AUC values are in line with expectation (Figure 4I), suggesting our nomogram had a robust prognostic predictive ability. In addition, we also compared the predictive sensitivity of angiogenesis subtype A/B, angiogenesis gene cluster A/B, and ARG score high/low to prognosis, and the results showed that ARGs score is the most sensitive to predict the prognosis of patients (Supplementary Figure S6).

### 3.6 The histological expression and mRNA expression of angiogenesis-associated risk genes

**FIGURE 5:**
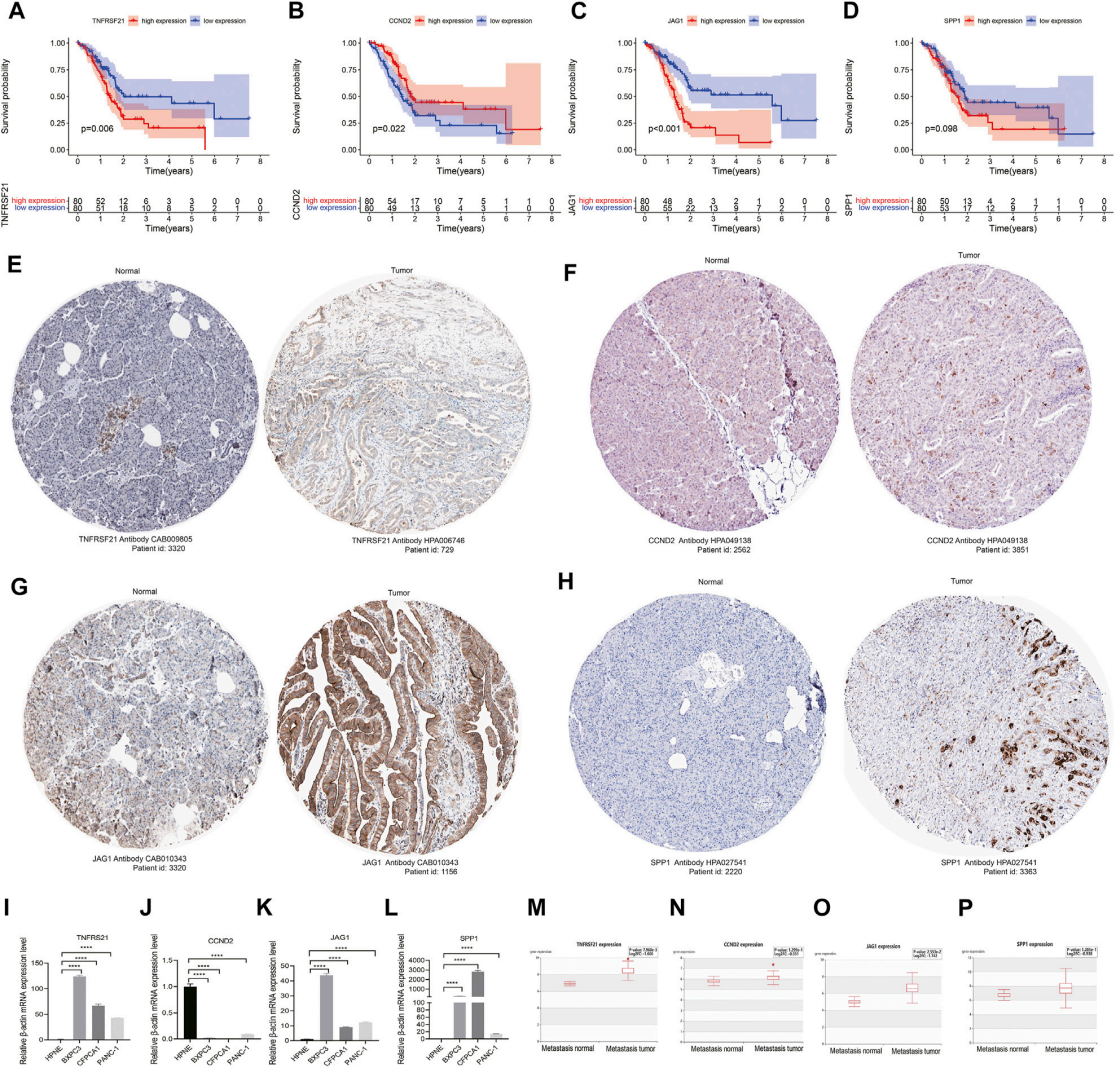
Validation of angiogenesis-associated risk genes in PC: **(A–D)** Survival analyses of four signature genes; **(F–H)** The histological expression of four signature genes from HPA database. The top of the figure indicates the category of tissue specimen. The name of angiogenesis gene, the antibody type used in immunohistochemistry, and the patient ID of tissue specimens are shown at the bottom of each image; **(I–L)** qRT-PCR analyses of the mRNA expression levels of angiogenesis-associated risk genes in PC cell lines (BXPC-3, CFPAC-1, and PANC-1) and normal pancreas cells (HPNE). **(M–P)** Correlation of angiogenesis-associated risk genes with tumor metastasis in PC. ****p < 0.0001 by repeated measures with two-tailed unpaired Student’s *t*-test.

The survival analysis indicated that the high expression of angiogenesis-related risk genes (*TNFRSF21* and *JAG1*) was associated with poor prognosis and high expression of *CCND2* was related to favorable survival (Figures 5A–D). In addition, significant difference of these genes in protein levels between pancreatic normal and cancer tissues was observed. Among that, the protein expression of *TNFRSF21, JAG1*, and *SPP1* were evidently up-regulated in tumor than normal tissues. However, discernable expressive difference was not found in *CCND2* (Figures 5E–H). Moreover, the mRNA expression of *TNFRSF21, JAG1*and *SPP1* was markedly elevated but *CCND2* was substantially declined in PC cells compared with normal pancreas cells (Figure 5I–L).

### 3.7 Correlation of angiogenesis-associated risk genes with tumor metastasis in PC

Metastasis is a critical factor for therapeutic resistance and poor patient survival. THCMDB tool was used to predict genes having metastatic potential. The results of HCMDB showed significantly higher expression level of *TNFRSF21, CCND2, JAG1*, and *SPP1* for patients with PC (Figures 5M–P), implying that these genes may promote PC metastasis.

### 3.8 Evaluation of TIME and ICPs between the high- and low-ARGs score groups

**FIGURE 6:**
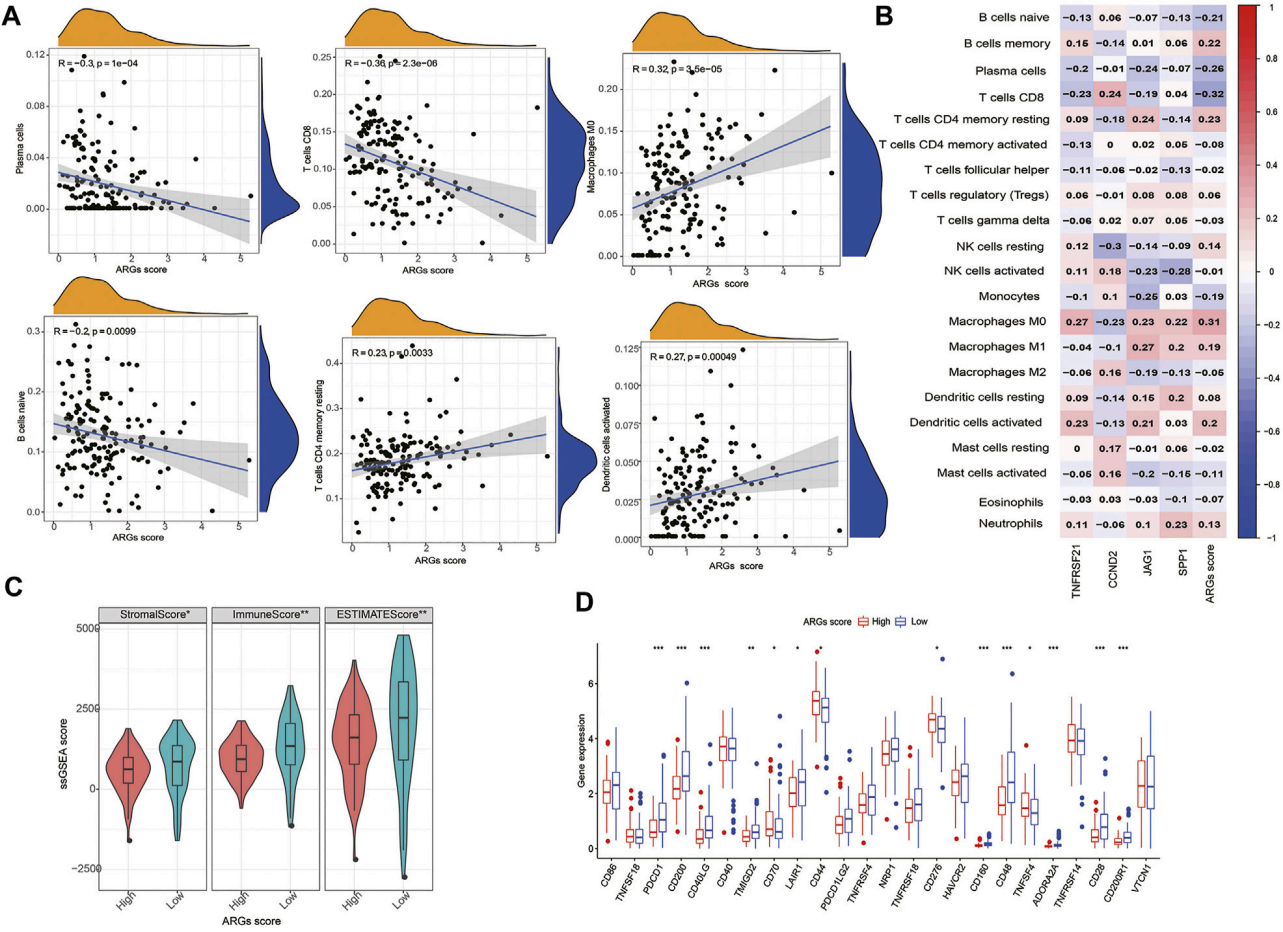
Evaluation of the TIME and checkpoints between the two groups: **(A)** Correlations between ARGs score and immune cell types; **(B)** Correlations between the abundance of immune cells and four genes in the proposed model; **(C)** Correlations between ARGs score and both immune and stromal scores; **(D)** Expression of immune checkpoints in the high and low-risk groups. ***p < 0.001, **p < 0.01, *p < 0.05 and not significant (p > 0.05) by repeated measures with the Wilcoxon test.

The infiltration of 22 immune cell subsets into the TME was further identified in the high- and low- ARGs score groups. Our results show that patients with low ARGs score are with higher immune score and more infiltrations of immune cells (CD8+T cells, activated memory CD4+T cells, plasma cells, naïve B cells, monocytes, and eosinophils) (Figures 6A,C). We also observed that most immune cells were significantly associated with the four hub biomarkers and ARGs score (Figure 6B). In addition, some ICPs (C*D200, CD40LG, CD70, CD44, TNFRSF18, CD276, CD160, TNFSF4*, and *VTCN1*) were differentially expressed in the two subgroups (Figure 6D).

### 3.9 Association of ARGs score with TMB and somatic mutations

**FIGURE 7:**
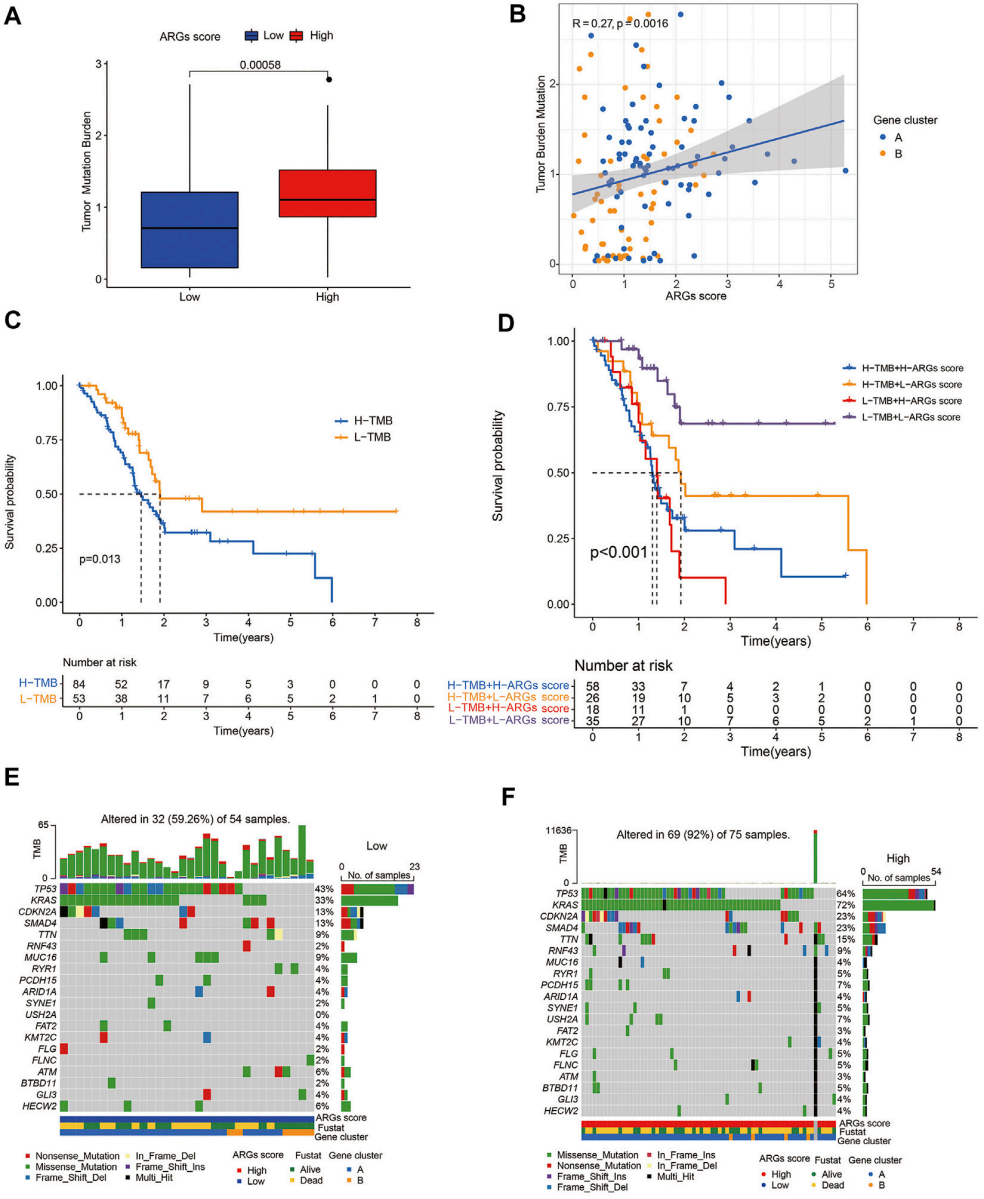
Association of ARGs score with TMB score in PC: **(A)** TMB in different ARGs score groups; **(B)** Spearman correlation analysis of the ARGs score and TMB; **(C)** Kaplan-Meier analysis of the OS between the low- and high-TMB groups; **(D)** Survival analysis among four patient groups stratified by both TMB and ARGs score; **(E,F)** The waterfall plot of somatic mutation features established with low and high ARGs score.

PC is a genetic disease. Somatic alterations in the most common driver genes (*KRAS, CDKN2A, TP53*, and *SMAD4*) play a pivotal role in PC biology and progression (Hayashi et al., 2021). To investigate the relationship of the ARGs score with somatic mutations, we compared the differences in TMB score and somatic mutations between the two groups. Our results indicated that low-TMB patients had a superior prognosis than high-TMB patients (Figure 7C), and the ARGs score was positively related to TMB score (Figures 7A,B). Additionally, the prognostic benefit of low TMB group was eliminated by high ARGs score (Figure 7D). Subsequently, we analyzed the distribution variations of the somatic mutations between two ARGs score groups in the TCGA-PAAD cohort. The top ten mutated genes in the high- and low-ARGs score groups were *TP53, KRAS, CDKN2*, *SMAD4, TTN, RNF43, MUC16, RYR1, PCDH15,* and *ARID1A*. Compared with patients with low ARGs scores, patients with high ARGs scores had dramatically higher frequencies of *TP53, KRAS*, *CDKN2, SMAD4*, and *TTN* mutations (Figures 7E,F).

### 3.10 Clinical outcomes and drug sensitivity of different ARGs score groups

**FIGURE 8:**
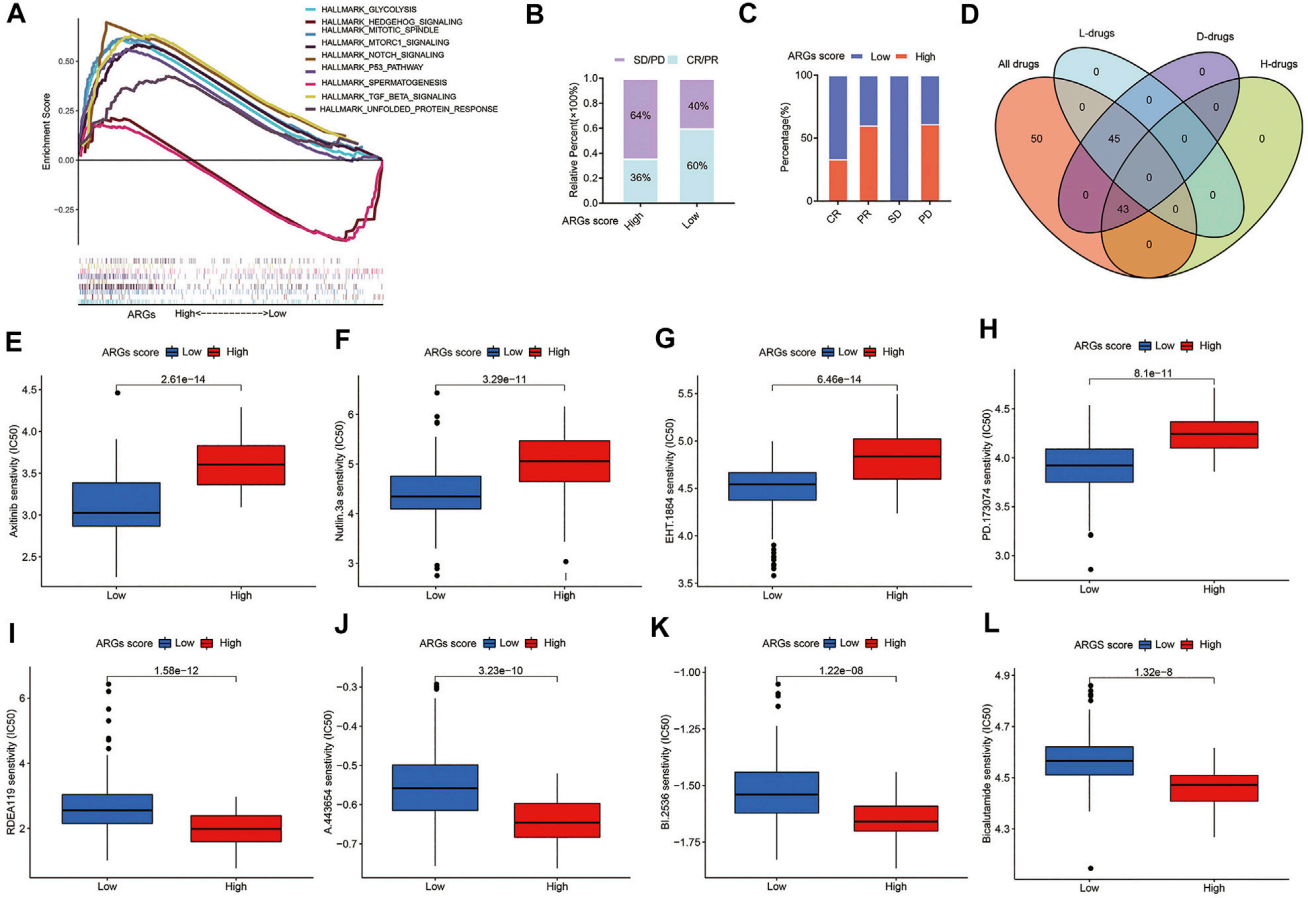
Relationships between ARGs score and therapeutic sensitivity: **(A)** GSEA analyzed the biological pathways of two ARGs score groups in the PC cohort; **(B)** The ratio of worse outcomes after surgery is greatly elevated in the high ARGs score group; **(C)** The proportion of clinical outcomes in PC patients with high and low ARGs score after surgery; **(D)** Venn diagram for summarizing included compounds from GDSC datasets; **(E–H)** The top 4 chemotherapeutic drugs PC patients with low ARGs score group are more susceptible to; **(I–L)** The top 4 chemotherapeutic drugs PC patients with high ARGs score group are more susceptible to. H, High ARGs score; L, Low ARGs score; D, differentiated.

The GSEA enrichment analysis shows that the high ARGs score group was mainly enriched in some pathways of tumorigenesis, such as the MTORC1, P53, and TGF-BETA signaling pathways (Figure 8A), implying that ARGs may play a crucial role in modulating tumor progression. Furthermore, patients with the low ARGs score had a higher CR/PR rate and a lower SD/PD rate after surgical therapy (Figures 8B,C).

For patients with advanced PC, chemotherapy and targeted therapy are the main treatments (Vincent et al., 2011). To confirm the efficacy of ARGs score as a biomarker to predict chemotherapy response in PC patients, we assessed the IC50 values of 138 chemotherapeutic drugs from GDSC dataset in TCGA-PAAD patients (Figure 8D). We found that there are 45 drugs patients with low ARGs score are more sensitive to (*p* < 0.05; Supplementary Table S8), including the top 2 chemotherapeutic drugs (Axitinib, EHT. 1864) and several targeted therapeutic drugs (Nutlin.3a and PD.173074, etc.) (Figures 8E–H). While there were 43 drugs that patients with high ARGs score may respond better to (*p* < 0.05; Supplementary Table S9), including the top 4 chemotherapeutic drugs (RDEA119, A.443,654, BI.2536, and Bicalutamide) (Figure 8I–L). In conclusion, these findings imply that patients with different ARGs score have distinct differences in sensitivity to chemotherapeutic drug therapy, and drugs of personalized therapy can be selected according to ARGs score.

## 4 Discussion

Due to high heterogeneity of PC, PC patients’ resistance to chemotherapy was common and their treatment outcome was extremely poor (Sipos et al., 2014; Ilic and Ilic, 2016). Despite progress in chemotherapy in recent years, guide of traditional histopathological classification to anti-tumor therapy may lead to difficulty in developing more specific and less resistant therapy (Springfeld et al., 2019). Several molecular classifications of PC were explored in previous studies, including classification based on single genetic markers including BRCA1/BRCA2, KRAS, TP53, ERBB2, and BRAF mutation, genomic aberrations patterns, or transcriptome profiling (Collisson et al., 2019). However, considerable heterogeneity has not been fully detected yet (Biankin et al., 2009; Hudson Chairperson et al., 2010; Collisson et al., 2019). A more accurate and clinically useful molecular classification of PC is urgently required to guide clinical practice.

Angiogenesis, a hallmark of cancer vital for growth and metastasis of multiple solid tumors, including PC (Baeriswyl and Christofori, 2009; Sajib et al., 2018; Annese et al., 2020), was found indispensable in regulating TIME in PC(Rivera and Bergers, 2015; Zhang et al., 2018; Rahma and Hodi, 2019). TIME is regarded as an important factor in tumor progression and immunotherapy response (Hinshaw and Shevde, 2019; Kuwahara et al., 2019; Sunami and Kleeff, 2019; Schizas et al., 2020), where higher lymphocyte infiltration indicated a more favorable prognosis (Governa et al., 2017; Kuwahara et al., 2019; Ma et al., 2020). Therefore, it can be surmised that anti-angiogenesis may boost immunotherapy (Rivera and Bergers, 2015; Trenti et al., 2018).

Thus, we explored the comprehensive role of ARGs in PC phenotype and TIME, and identified two angiogenesis clusters based on the prognostic 11 ARGs. Significant differences lie in clinical outcomes and TIME between the two clusters. Additionally, ARGs score, an angiogenesis-related gene signature was constructed to recognize the angiogenesis molecule classification for predicting TIME and prognosis in PC. Patients with low and high ARGs scores displayed diverse prognosis, TMB, TIME, ICPs, and drug sensibility. Moreover, by combining ARGs score with other clinical variables, we established a quantitative nomogram to further improve application of ARGs score.

In our study, angiogenesis subtype A characterized by immunosuppression was linked to a higher ARGs score. Angiogenesis cluster B featured by immune cell activation was linked to a lower ARGs score. More interestingly, higher enrichment of B cells, plasma cells, CD8+T cells, and monocytes were observed in low ARGs score group, promoting anti-cancer immunity. Whereas, higher enrichment of CD4+memory resting T cells and macrophage M0 cells were observed in high ARGs score group. CD4+T cells and macrophages were found negative and complex in tumor immunity (Ribatti and Crivellato, 2009; Saito et al., 2016; Dehne et al., 2017), respectively. These findings further support that high ARGs scores should be linked to immunosuppression and poor prognosis. Therefore, ARGs might play a crucial role in TIME and progression in PC. As revealed in several studies, angiogenesis factors might operate as immune modulators, causing pathological vascularization and thereby contributing to tumorigenesis (Ribatti and Crivellato, 2009; Minton, 2019).

Immune checkpoint inhibitors (ICIs) have become an anti-tumor treatment trend (Bagchi et al., 2021; Lentz et al., 2021). We observed that expression of various ICPs, including CD200, CD40LG, CD44, TNFRSF18, CD160, TNFSF4, and VTCN1, are up-regulated in low ARGs score group, implying that patients in this group might benefit from these ICIs. Thus, targeting ARGs may be beneficial for PC immunotherapy. Accordingly, we further recognized the latent susceptible chemotherapeutic and targeted drugs in patients with different ARGs scores. Some drugs, including Axitinib, EHT.1864, Nutlin.3a, and PD.173074, and some other drugs, including RDEA119, A.443654, BI.2536, and Bicalutamide were identified for low- and high-ARGs score PC patients, respectively. Combination of these drugs with anti-angiogenesis may help alleviate drug resistance and improve survival in PC patients. Axitinib is vascular endothelial growth factor receptor tyrosine kinase inhibitor, efficacy of which was evaluated in phase II/III studies of patients in many tumor types including pancreatic cancer (Kelly and Rixe, 2010; Grünwald et al., 2020). RDEA119, an allosteric MEK inhibitor, has been selected for clinical development because of its potency and favorable pharmacokinetic profile (Iverson et al., 2009). Moreover, RDEA119 combined with rapamycin showed significant anti-tumor activity in human orthotopic primary PC xenografts (Chang et al., 2010).

Overall, this study not only sheds light on personalized prediction approaches, but also provides clues for precision therapy in PC. ARGs score model has significant clinical significance in both low- and high-ARGs score patients. For patients with high ARGs scores, we offered the potential drugs to effectively improve their prognosis. For patients with low ARGs scores, clinicians could adopt ICIs immunotherapy and some targeted drugs in our study to improve prognosis of PC patients.

Some limitations to our study should be acknowledged. First, data from public databases are obtained retrospectively, so the prognostic robustness and clinical usefulness of the angiogenesis-related gene signature need further validation in larger prospective trials. Second, further vivo and *in vitro* experimental studies are necessary to gain insight into the relationship between ARGs scores and TME, thus confirming our findings in PC.

## Data Availability

The datasets presented in this study can be found in online repositories. The names of the repository/repositories and accession numbers can be found in the article/Supplementary Material.

## Glossary

ARGs: angiogenesis-related genes
CR: Complete Response
DEGs: differentially expressed genes
EMT: epithelial-mesenchymal transition
FC: fold change
GDSC: Genomics of Drug Sensitivity in Cancer
GSEA: gene set enrichment analysis
GSVA: gene set variation analysis
GTEx: Genotype-Tissue Expression
HPA: Human Protein Atlas
IC50: half-maximal inhibitory concentration
ICGC: International Cancer Genome Consortium
ICIs: immune checkpoint inhibitors
ICP: immune checkpoint
LASSO: least absolute shrinkage and selection operator
MAF: mutation annotation format
MSigDB: Molecular Signatures Database
OS: overall survival
PC: pancreatic cancer
PCA: principal component analysis
PD: Progressive Disease
PR: Partial Response;
SD: Stable Disease
qRT-PCR: quantitative real-time PCR reaction.
ROC: receiver operating characteristic
ssGSEA: single sample gene set enrichment analysis
TCGA: The Cancer Genome Atlas
TIIC: tumor-infiltrating immune cells
TIME: tumor immune microenvironment
TMB: tumor mutation burden
TME: tumor microenvironment
tROC: time-dependent receiver operating characteristic
