# Supplementary material for: A novel angiogenesis-based molecular signature related to prognosis and tumor immune interactions of pancreatic cancer
Source: Front Cell Dev Biol. 2022 Oct 6;10:1001606. doi: 10.3389/fcell.2022.1001606 (PMC9582445; doi:10.3389/fcell.2022.1001606)
Supplement: Supplementary file 1 [file DataSheet1.zip › Supplementary_Material(1).docx]

Supplementary Material

# Supplementary Figures

**Supplementary Figure 1.** The flow chart of this study.

**Supplementary Figure 2.** A consensus clustering algorithm to categorize the patients with PC based on the expression profiles of the 11 ARGs.

**Supplementary Figure 3.** The two distinct subtypes identified by consensus clustering analysis were again clearly identified in PC patients from ICGC cohort.

**Supplementary Figure 4.** Identification of gene subtypes based on DEGs: (A) GO enrichment analyses of DEGs among two angiogenesis subtypes; (B) Relationships between clinical features and the two gene subtypes; (C) PCA analysis of 332 DEGs in the TCGA cohort identified two distinct gene clusters; (D) Kaplan-Meier survival curve showed significant differences among the two gene clusters in the TCGA cohort; (E) Expression of 11 angiogenesis genes in two gene clusters.

DEGs, differentially expressed protein-coding genes; GO, Gene Ontology; FDR, False Discovery Rate; ***p < 0.001, **p < 0.01, *p < 0.05 and not significant (p > 0.05) by repeated measures with the Wilcoxon test.

**Supplementary Figure 5.** Expression patterns of four selected prognostic biomarkers in high- and low-ARGs score groups.

**Supplementary Figure 6.** The time−dependent ROC curves of the nomograms compared for 1−, 2−, and 3−year OS in PC, respectively.

# Supplementary Tables

**Supplementary Table 1.** The primers of four selected prognostic biomarkers in high- and low-ARGs score groups.

**Supplementary Table 2.** Detailed information on the 160 PC patients from TCGA cohort.

**Supplementary Table 3.** Univariate Cox regression analysis showing the prognostic values of 11 ARGs in patients with PC.

**Supplementary Table 4.** The comprehensive landscape of ARGs interactions, regulator connections, and their prognostic value in PC patients.

**Supplementary Table 5.** GSVA enrichment analysis of the angiogenesis subtypes on TCGA and ICGC cohorts.

**Supplementary Table 6.** 332 angiogenesis subtype-related DEGs in TCGA cohort.

**Supplementary Table 7.** The correlation the ARGs score and four key biomarkers.

**Supplementary Table 8.** 45 drugs are highly sensitive to patients with low ARGs score.

**Supplementary Table 9.** 43 drugs are highly sensitive to patients with high ARGs score.
