# Supplementary figures and images for: A novel angiogenesis-based molecular signature related to prognosis and tumor immune interactions of pancreatic cancer
Source: Front Cell Dev Biol. 2022 Oct 6;10:1001606. doi: 10.3389/fcell.2022.1001606 (PMC9582445; doi:10.3389/fcell.2022.1001606)

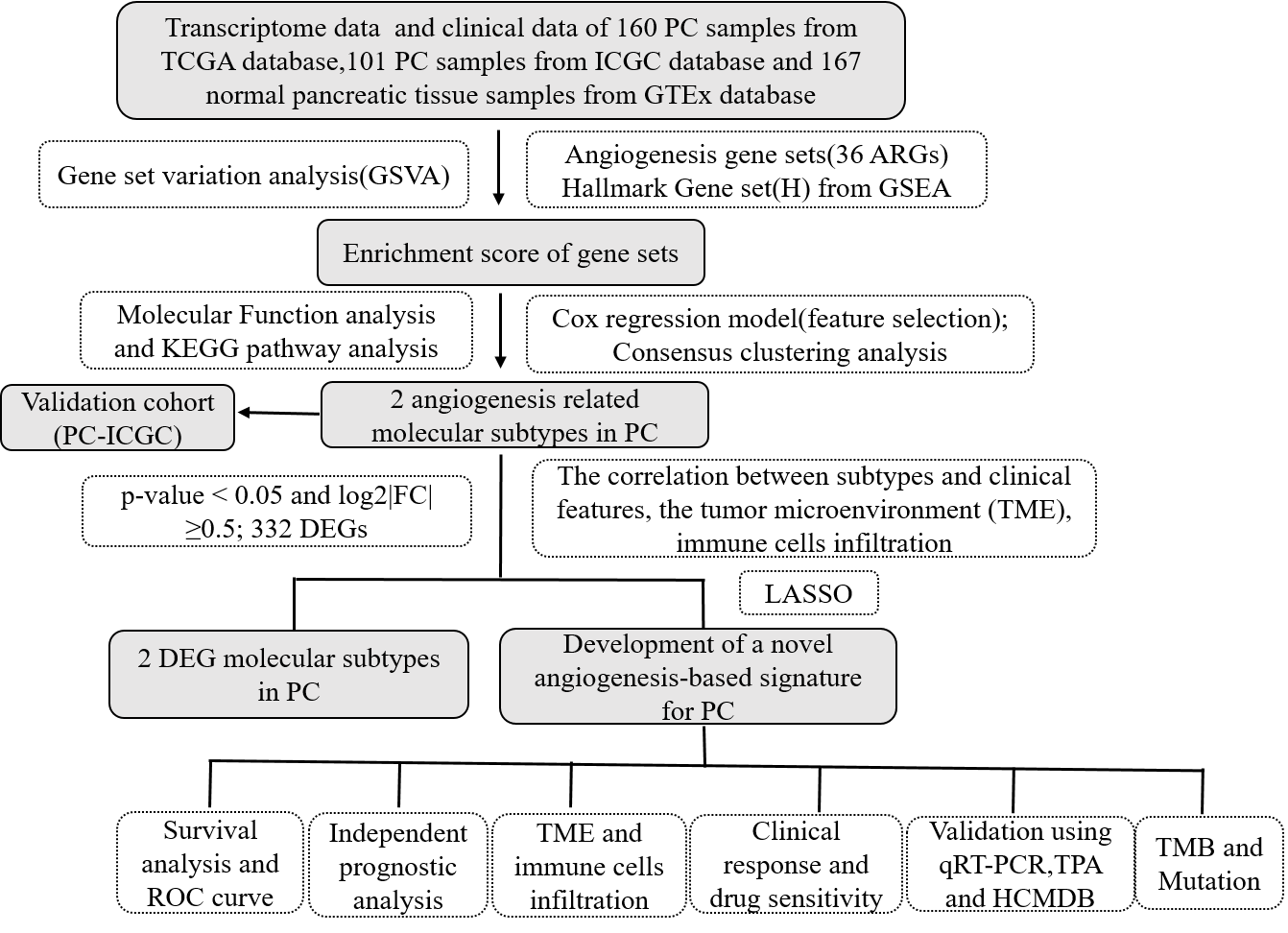

Supplement: Supplementary file 1 [file DataSheet1.zip › Supplementary Figure S1.tif]

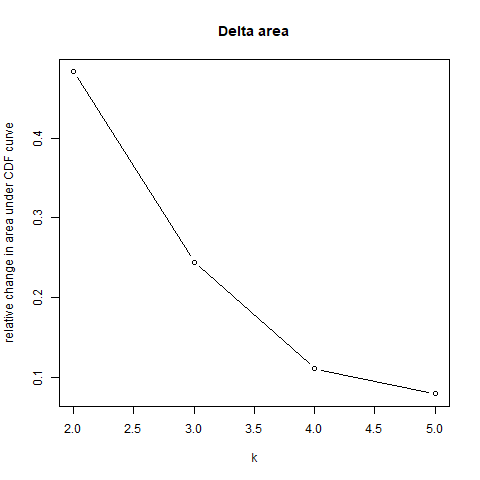

Supplement: Supplementary file 1 [file DataSheet1.zip › Supplementary Figure S2.png]

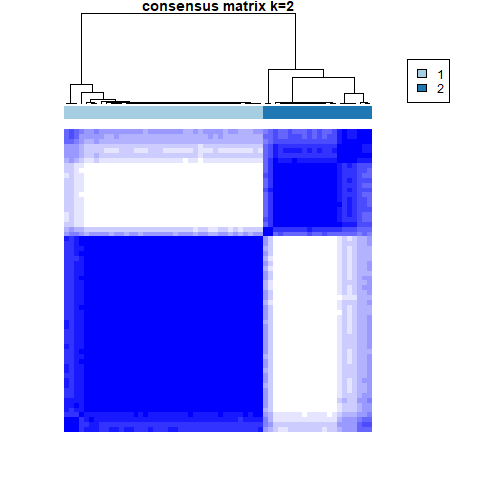

Supplement: Supplementary file 1 [file DataSheet1.zip › Supplementary Figure S3.png]

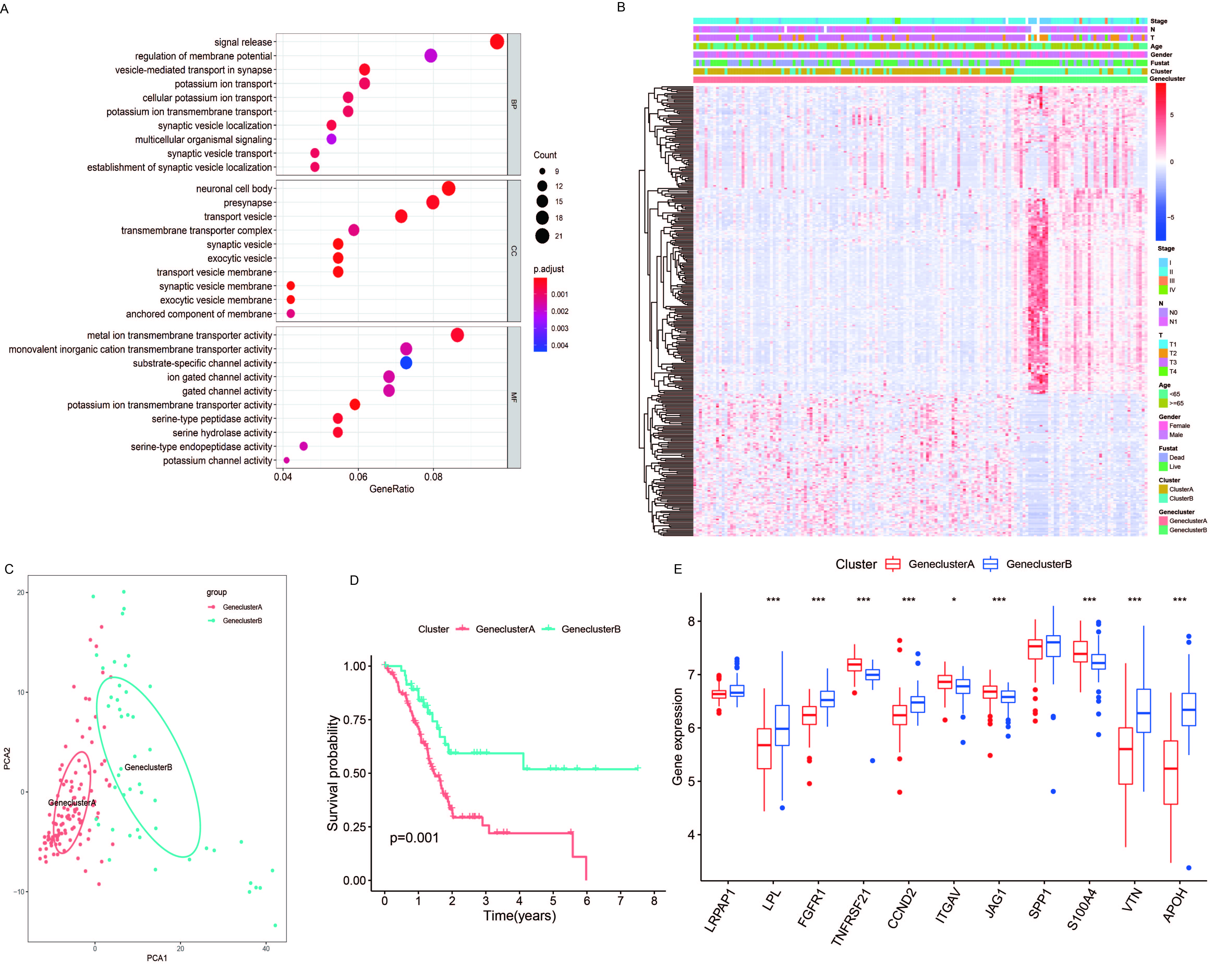

Supplement: Supplementary file 1 [file DataSheet1.zip › Supplementary Figure S4.jpg]

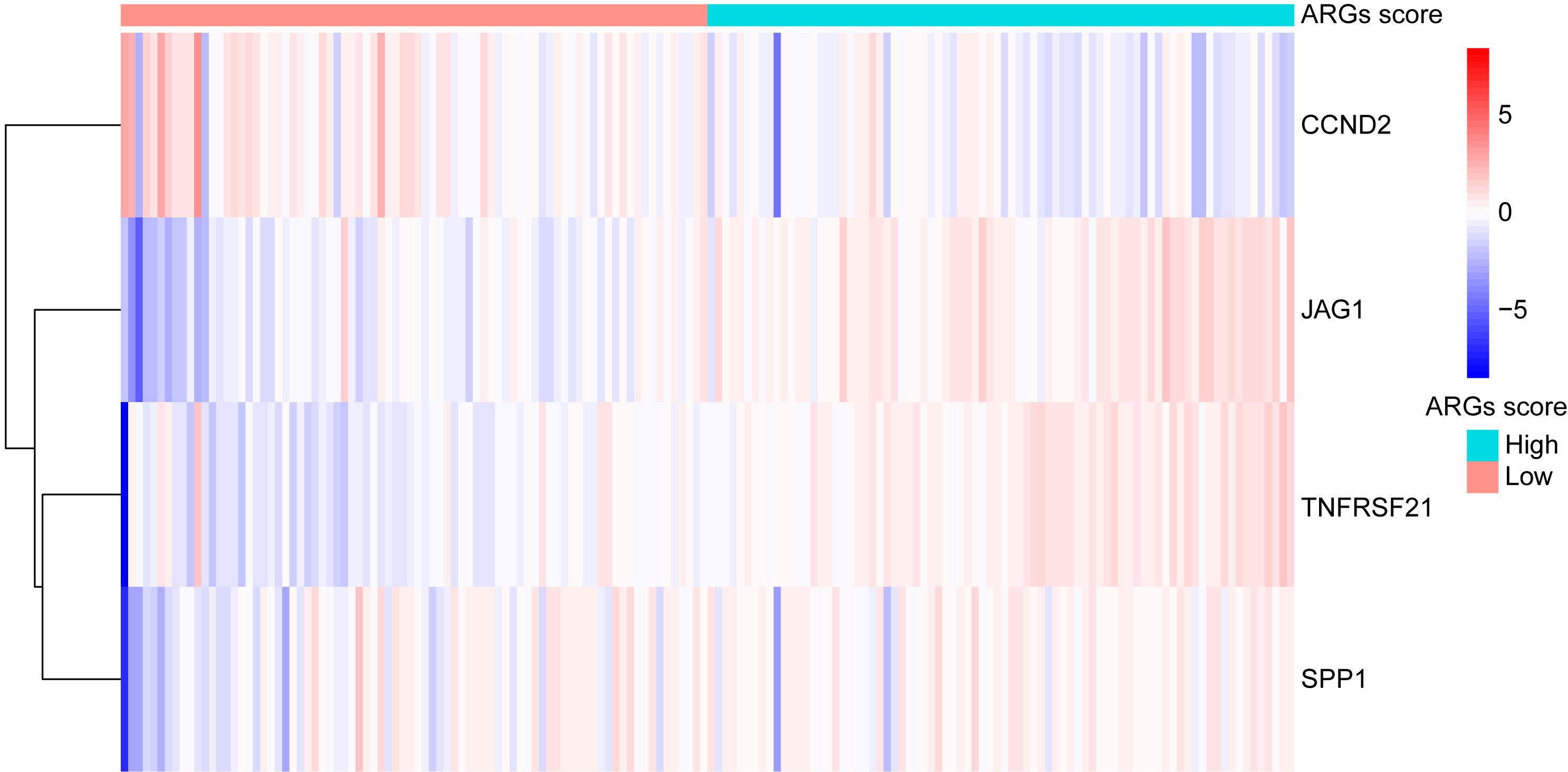

Supplement: Supplementary file 1 [file DataSheet1.zip › Supplementary Figure S5.jpg]

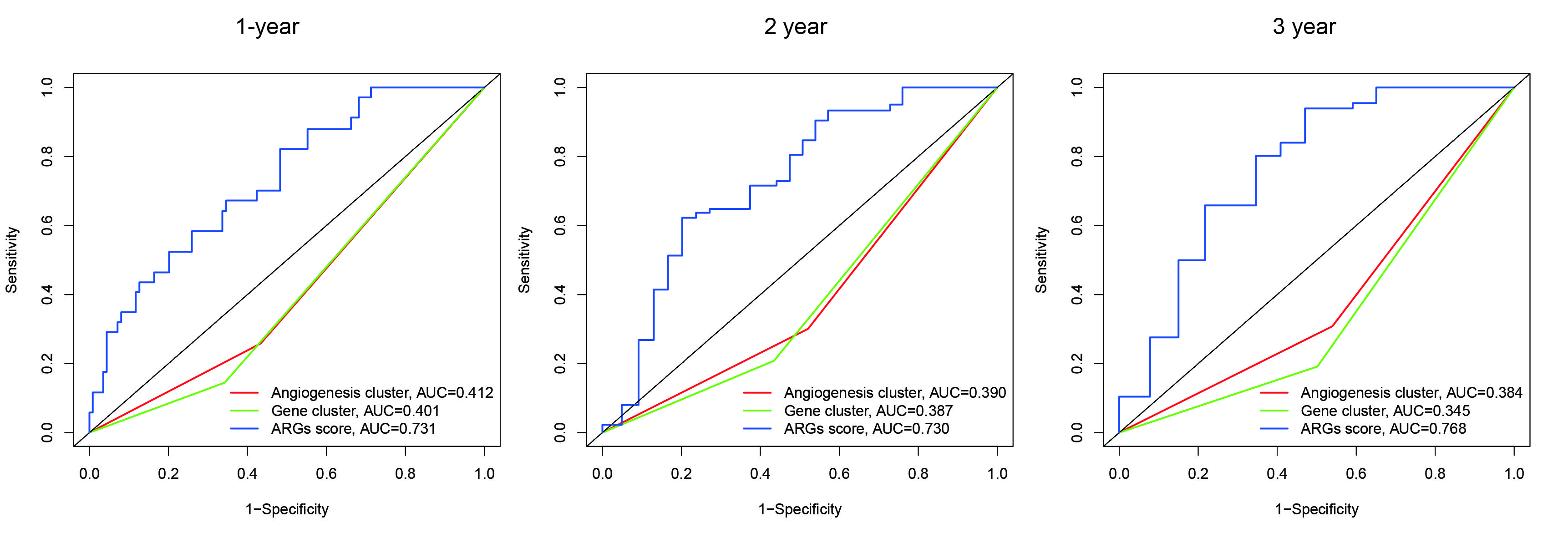

Supplement: Supplementary file 1 [file DataSheet1.zip › Supplementary Figure S6.jpg]
